# 3-Phenyl­tetra­hydro­furan-2,5-dione

**DOI:** 10.1107/S1600536808042670

**Published:** 2008-12-20

**Authors:** Li Quan, Handong Yin

**Affiliations:** a College of Chemistry and Chemical Engineering, Liaocheng University, Shandong 252059, People’s Republic of China

## Abstract

In the title compound, C_10_H_8_O_3_, the dihedral angle between the approximately planar tetra­hydro­furan-2,5-dione ring [maximum deviation 0.014 (3) Å] and the phenyl ring is 85.68 (8)°. Weak C—H⋯O=C inter­molecular hydrogen-bonding contacts are observed in the structure.

## Related literature

For the crystal structure of the related compound, 3,3-dimethyl-4-phenyl­tetra­hydro­furan-2,5-dione, see: Rudler *et al.* (2005 ▶). For hydrogen bonds, see: Desiraju & Steiner (2001 ▶); Jeffrey & Saenger (1994 ▶).
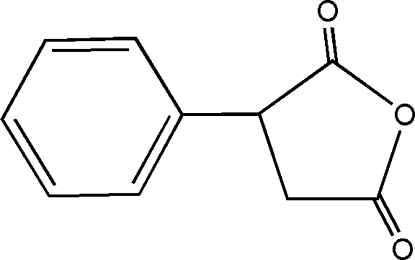


## Experimental

### 

#### Crystal data

C_10_H_8_O_3_
*M* *_r_* = 176.16
Orthorhombic, 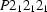

*a* = 5.6172 (9) Å
*b* = 10.1460 (12) Å
*c* = 14.9899 (19) Å
*V* = 854.3 (2) Å^3^
*Z* = 4
Mo *K*α radiation
μ = 0.10 mm^−1^
*T* = 298 (2) K
0.43 × 0.18 × 0.15 mm

#### Data collection

Siemens SMART diffractometer
Absorption correction: multi-scan (*SADABS*; Sheldrick, 1996 ▶) *T*_min_ = 0.958, *T*_max_ = 0.985
4082 measured reflections
905 independent reflections
583 reflections with *I* > 2σ(*I*)
*R*_int_ = 0.048

#### Refinement

*R*[*F* ^2^ > 2σ(*F* ^2^)] = 0.035
*wR*(*F* ^2^) = 0.069
*S* = 1.14
905 reflections
124 parameters
4 restraints
H atoms treated by a mixture of independent and constrained refinement
Δρ_max_ = 0.11 e Å^−3^
Δρ_min_ = −0.12 e Å^−3^

### 

Data collection: *SMART* (Siemens, 1996 ▶); cell refinement: *SAINT* (Siemens, 1996 ▶); data reduction: *SAINT*; program(s) used to solve structure: *SHELXS97* (Sheldrick, 2008 ▶); program(s) used to refine structure: *SHELXL97* (Sheldrick, 2008 ▶); molecular graphics: *ORTEP-3* (Farrugia, 1997 ▶) and *PLATON* (Spek, 2003 ▶); software used to prepare material for publication: *SHELXTL* (Sheldrick, 2008 ▶) and *PLATON*.

## Supplementary Material

Crystal structure: contains datablocks I, global. DOI: 10.1107/S1600536808042670/si2139sup1.cif

Structure factors: contains datablocks I. DOI: 10.1107/S1600536808042670/si2139Isup2.hkl

Additional supplementary materials:  crystallographic information; 3D view; checkCIF report

## Figures and Tables

**Table 1:** Hydrogen-bond geometry (Å, °)

*D*—H⋯*A*	*D*—H	H⋯*A*	*D*⋯*A*	*D*—H⋯*A*
C3—H3*B*⋯O3^i^	1.02 (2)	2.60 (2)	3.446 (4)	140 (2)
C8—H8⋯O2^ii^	1.00 (2)	2.65 (2)	3.409 (4)	133 (2)
C8—H8⋯O3^iii^	1.00 (2)	2.58 (2)	3.373 (4)	136 (2)

Symmetry codes: (i) 
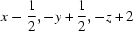
; (ii) 
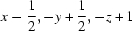
; (iii) 
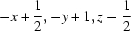
.

**Fig. 1.**
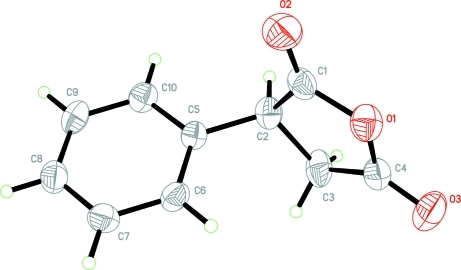
The molecular structure of the title compound, showing displacement ellipsoids drawn at the 30% probability level.

**Fig. 2.**
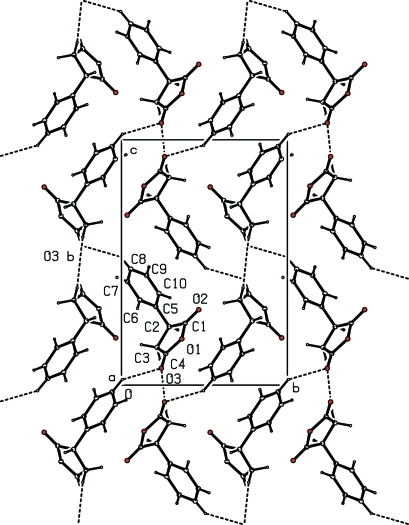
A projection of the title compound (I) viewed down the a axis. Weak C—H···O contacts are indicated as dashed lines.

**Fig. 3.**
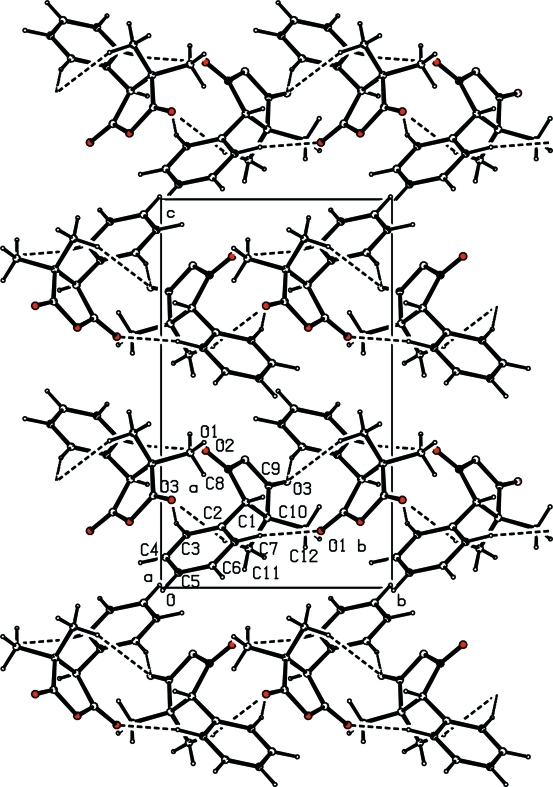
A section of the structure of (II) viewed down the a axis (II = 3,3-dimethyl-4-phenyltetrahydrofuran-2,5-dione). The C—H···O bonds extending along the b axis are shown as dashed lines.

## supplementary crystallographic information

### Comment

Initially, the structure of the title compound (I) was refined with an absolute structure parameter x (Flack, 1983) of 0.0(1.9), which is a meaningless result. As a consequence, the Friedel pairs were averaged. Thus, the absolute structure of the title compound (Fig.1) is unknown and the chiral atom C2 indicates the S* form (Fig. 1). A similar compound, 3,3-dimethyl-4-phenyltetrahydrofuran-2,5-dione, (Rudler *et al.* 2005) crystallized in the centrosymmetric space group P2_1_/n, with racemic forms R* and S* in the structure.

Normally, a twist or envelope form for the THF-2,5-dione ring was expected. In the title structure, the 2,5-dione ring is essentially planar, with the chiral atom C2 within the plane, whereas in the 3,3-dimethyl-2,5-dione ring (Rudler *et al.* 2005), a flattened envelope form was observed, with the chiral atom C1 being slightly out-of-plane. Interestingly, the title molecule has a dihedral angle of 85.68 (8)° between the phenyl ring and the planar tetrahydrofurane-2,5-dione ring.

The dione C==O groups are normally good acceptors for intermolecular weak C—H···O contacts in the absence of classic donors (O–H, N–H). In the title structure, the C—H···O==C contacts should be considered as very weak interactions. Two H···O distances are below the accepted maximum values of 2.65 - 2.66 Å which are reported in the literature (Jeffrey & Saenger, 1994, p. 157). Weak intra- and intermolecular hydrogen bonds are also extensively discussed, with many structural examples, by Desiraju & Steiner (2001).

For the following comparison of the title structure (I) and the related structure reported by Rudler *et al.* (2005) (II), the CIF of (II) has been requested from the Cambridge Crystallographic Data Centre (CCDC) by using the assigned CCDC No. 266338. Calculation of geometric details for both structures and for preparing Figures 2 and 3, the programme PLATON (Spek, 2003) was used, including the check.CIF procedures. Inspection of the hydrogen bond geometry in the 3,3-dimethyl analogue structure (II) (Rudler *et al.* 2005) however, with C–H distances 1.00 - 1.03 Å, showed acceptable C—H···O==C bonds. For a fair comparison of both structures, hard distance restraints (DFIX 1.02 (0.02) Å) for C8–H8 and C3–H3B were applied in the re-refinement of the title structure. As a result, two of the three intermolecular contacts C—H···O==C (Table 1) with O3 as a bifurcated acceptor, showed up to form a three-dimensional hydrogen bonding network, due to the screw  axes (2_1_) distribution in the cell (Fig. 2). Interestingly, in the dimethyl-structure (II), the molecules are linked by weak intermolecular C—H···O==C hydrogen bonding contacts to form layers along the *b* axis (Fig. 3). The intermolecular C—H···O hydrogen bonding contacs in (II) have shorter H···O distances and larger angles around the H atoms, and one of the methyl groups is a donor. The calculated H···O distances are 2.36, 2.44 and 2.53 Å, the corresponding angles are 170, 162 and 159 °. These contacts are much stronger than those observed in the title compound (I).

### Experimental

Pyrazine-2,3-dicarboxylic acid ( 0.336 g, 2 mmol) was added to stirring toluene solution (25 ml) containing triphenylantimonyoxide (0.738 g, 2 mmol). After refluxing for 8 h, the solution was filtered. The solvent was gradually removed by evaporation under vacuum until the white solid is obtained. The solid was recrystallized from petroleum ether/dichoromethane (1:1) to give colorless crystals.

### Refinement

The H atom bound to the (phenyl) ring was constraint to values of 0.93Å, the CH and CH_2_ groups were 0.98Å and 0.97Å with *U*_iso_(H) = 1.2 *U*_eq_. The phenyl H atom, H8, and one of the CH_2_ H atoms, H3B, were refined using distance restraints (DFIX 1.02 (0.02) Å, see Table 1) for comparison with similar C—H···O hydrogen bonds (C—H = 1.00 - 1.03 Å) in the related structure (II) (but in centrosymmetric space group P2_1_/n).

In the absence of significant anomalous dispersion effects, Friedel pairs were averaged, with the result of a poor data/parameter ratio of 7.67.

### Crystal data

**Table:**

C_10_H_8_O_3_	*D*_x_ = 1.370 Mg m^−^^3^
*M**_r_* = 176.16	Mo *K*α radiation, λ = 0.71073 Å
Orthorhombic, *P*2_1_2_1_2_1_	Cell parameters from 826 reflections
*a* = 5.6172 (9) Å	θ = 2.7–29.9°
*b* = 10.1460 (12) Å	µ = 0.10 mm^−^^1^
*c* = 14.9899 (19) Å	*T* = 298 K
*V* = 854.3 (2) Å^3^	Block, colorless
*Z* = 4	0.43 × 0.18 × 0.15 mm
*F*(000) = 368	

### Data collection

**Table:**

Siemens SMART diffractometer	905 independent reflections
Radiation source: fine-focus sealed tube	583 reflections with *I* > 2σ(*I*)
graphite	*R*_int_ = 0.048
φ and ω scans	θ_max_ = 25.0°, θ_min_ = 2.4°
Absorption correction: multi-scan (*SADABS*; Sheldrick, 1996)	*h* = −6→6
*T*_min_ = 0.958, *T*_max_ = 0.985	*k* = −12→9
4082 measured reflections	*l* = −15→17

### Refinement

**Table:**

Refinement on *F*^2^	Primary atom site location: structure-invariant direct methods
Least-squares matrix: full	Secondary atom site location: difference Fourier map
*R*[*F*^2^ > 2σ(*F*^2^)] = 0.035	Hydrogen site location: inferred from neighbouring sites
*wR*(*F*^2^) = 0.069	H atoms treated by a mixture of independent and constrained refinement
*S* = 1.14	*w* = 1/[σ^2^(*F*_o_^2^) + (0.0218*P*)^2^] where *P* = (*F*_o_^2^ + 2*F*_c_^2^)/3
905 reflections	(Δ/σ)_max_ < 0.001
124 parameters	Δρ_max_ = 0.11 e Å^−^^3^
4 restraints	Δρ_min_ = −0.12 e Å^−^^3^

### Special details

**Table:**

Geometry. All esds (except the esd in the dihedral angle between two l.s. planes) are estimated using the full covariance matrix. The cell esds are taken into account individually in the estimation of esds in distances, angles and torsion angles; correlations between esds in cell parameters are only used when they are defined by crystal symmetry. An approximate (isotropic) treatment of cell esds is used for estimating esds involving l.s. planes.
Refinement. Refinement of F^2^ against ALL reflections. The weighted R-factor wR and goodness of fit S are based on F^2^, conventional R-factors R are based on F, with F set to zero for negative F^2^. The threshold expression of F^2^ > 2sigma(F^2^) is used only for calculating R-factors(gt) etc. and is not relevant to the choice of reflections for refinement. R-factors based on F^2^ are statistically about twice as large as those based on F, and R- factors based on ALL data will be even larger.

### Fractional atomic coordinates and isotropic or equivalent isotropic displacement parameters (Å^2^)

**Table:**

	*x*	*y*	*z*	*U*_iso_*/*U*_eq_	
O1	0.4229 (4)	0.1359 (2)	0.81130 (15)	0.0641 (6)	
O2	0.2752 (5)	0.0346 (2)	0.69260 (15)	0.0814 (8)	
O3	0.4849 (4)	0.2600 (3)	0.93134 (16)	0.0959 (10)	
C1	0.2504 (7)	0.1168 (3)	0.7480 (2)	0.0538 (8)	
C2	0.0469 (6)	0.2115 (3)	0.76195 (17)	0.0539 (8)	
H2	−0.0975	0.1607	0.7747	0.065*	
C3	0.1200 (7)	0.2861 (3)	0.84658 (19)	0.0657 (10)	
H3A	0.1304	0.3800	0.8349	0.079*	
H3B	0.001 (4)	0.267 (3)	0.8964 (14)	0.079*	
C4	0.3563 (7)	0.2335 (3)	0.8716 (2)	0.0608 (9)	
C5	0.0043 (5)	0.2936 (3)	0.67943 (17)	0.0450 (7)	
C6	0.1636 (5)	0.3888 (3)	0.65339 (19)	0.0525 (8)	
H6	0.3001	0.4032	0.6871	0.063*	
C7	0.1241 (7)	0.4634 (3)	0.5779 (2)	0.0623 (9)	
H7	0.2342	0.5271	0.5611	0.075*	
C8	−0.0773 (7)	0.4438 (3)	0.5275 (2)	0.0614 (9)	
H8	−0.115 (5)	0.505 (2)	0.4773 (14)	0.074*	
C9	−0.2372 (6)	0.3485 (3)	0.55219 (19)	0.0626 (10)	
H9	−0.3733	0.3343	0.5182	0.075*	
C10	−0.1959 (5)	0.2734 (3)	0.62758 (19)	0.0550 (8)	
H10	−0.3043	0.2083	0.6436	0.066*	

### Atomic displacement parameters (Å^2^)

**Table:**

	*U*^11^	*U*^22^	*U*^33^	*U*^12^	*U*^13^	*U*^23^
O1	0.0586 (15)	0.0705 (14)	0.0630 (14)	0.0132 (13)	0.0002 (13)	0.0001 (13)
O2	0.115 (2)	0.0673 (15)	0.0614 (14)	0.0060 (15)	0.0136 (17)	−0.0120 (12)
O3	0.099 (2)	0.104 (2)	0.0843 (17)	−0.0020 (17)	−0.0379 (17)	−0.0119 (15)
C1	0.068 (2)	0.052 (2)	0.0422 (18)	0.001 (2)	0.009 (2)	0.0096 (17)
C2	0.048 (2)	0.0672 (18)	0.0463 (19)	0.0028 (19)	0.0053 (16)	0.0059 (17)
C3	0.081 (3)	0.079 (2)	0.0376 (18)	0.022 (2)	0.0030 (18)	0.0019 (17)
C4	0.073 (3)	0.059 (2)	0.050 (2)	0.001 (2)	−0.007 (2)	0.0065 (19)
C5	0.0393 (19)	0.0519 (18)	0.0438 (17)	0.0014 (17)	0.0004 (16)	0.0007 (15)
C6	0.045 (2)	0.063 (2)	0.0499 (19)	−0.0080 (18)	−0.0062 (16)	−0.0024 (16)
C7	0.072 (3)	0.055 (2)	0.060 (2)	−0.0102 (19)	0.003 (2)	0.0008 (18)
C8	0.078 (3)	0.057 (2)	0.049 (2)	0.010 (2)	−0.006 (2)	−0.0008 (16)
C9	0.054 (2)	0.081 (3)	0.053 (2)	0.004 (2)	−0.014 (2)	−0.0067 (17)
C10	0.043 (2)	0.068 (2)	0.0545 (19)	−0.0044 (19)	0.0001 (17)	−0.0010 (17)

### Geometric parameters (Å, °)

**Table:**

O1—C1	1.371 (3)	C5—C6	1.374 (3)
O1—C4	1.392 (3)	C5—C10	1.382 (4)
O2—C1	1.185 (3)	C6—C7	1.378 (4)
O3—C4	1.182 (3)	C6—H6	0.9300
C1—C2	1.508 (4)	C7—C8	1.375 (4)
C2—C5	1.510 (3)	C7—H7	0.9300
C2—C3	1.533 (4)	C8—C9	1.371 (4)
C2—H2	0.9800	C8—H8	1.00 (2)
C3—C4	1.479 (4)	C9—C10	1.383 (4)
C3—H3A	0.9700	C9—H9	0.9300
C3—H3B	1.02 (2)	C10—H10	0.9300
			
C1—O1—C4	111.1 (2)	C6—C5—C10	118.3 (3)
O2—C1—O1	120.1 (3)	C6—C5—C2	121.2 (3)
O2—C1—C2	129.4 (3)	C10—C5—C2	120.5 (3)
O1—C1—C2	110.5 (2)	C7—C6—C5	120.9 (3)
C1—C2—C5	110.9 (2)	C7—C6—H6	119.5
C1—C2—C3	103.1 (3)	C5—C6—H6	119.5
C5—C2—C3	116.6 (3)	C8—C7—C6	120.3 (3)
C1—C2—H2	108.6	C8—C7—H7	119.9
C5—C2—H2	108.6	C6—C7—H7	119.9
C3—C2—H2	108.6	C9—C8—C7	119.5 (3)
C4—C3—C2	105.8 (3)	C9—C8—H8	120.4 (16)
C4—C3—H3A	110.3	C7—C8—H8	119.7 (15)
C2—C3—H3A	110.6	C8—C9—C10	120.0 (3)
C4—C3—H3B	109.4 (14)	C8—C9—H9	120.0
C2—C3—H3B	109.6 (14)	C10—C9—H9	120.0
H3A—C3—H3B	111.1	C5—C10—C9	120.9 (3)
O3—C4—O1	119.3 (3)	C5—C10—H10	119.5
O3—C4—C3	131.2 (4)	C9—C10—H10	119.5
O1—C4—C3	109.5 (3)		

### Hydrogen-bond geometry (Å, °)

**Table:**

*D*—H···*A*	*D*—H	H···*A*	*D*···*A*	*D*—H···*A*
C3—H3B···O3^i^	1.02 (2)	2.60 (2)	3.446 (4)	140 (2)
C8—H8···O2^ii^	1.00 (2)	2.65 (2)	3.409 (4)	133 (2)
C8—H8···O3^iii^	1.00 (2)	2.58 (2)	3.373 (4)	136 (2)

Symmetry codes: (i) *x*−1/2, −*y*+1/2, −*z*+2; (ii) *x*−1/2, −*y*+1/2, −*z*+1; (iii) −*x*+1/2, −*y*+1, *z*−1/2.
